# A comparative study on the effectiveness of paracervical block and parenteral diclofenac for pain relief during manual vacuum aspiration

**DOI:** 10.4314/ahs.v23i3.4

**Published:** 2023-09

**Authors:** Iyke F Osinachi, Godwin O Akaba, Nathaniel D Adewole, Kate I Omonua, Bissallah A Ekele

**Affiliations:** 1 Department of Obstetrics and Gynaecology, University of Abuja/ University of Abuja Teaching Hospital, Abuja, Nigeria; 2 Department of Obstetrics and Gynaecology, University of Abuja Teaching Hospital, Abuja, Nigeria

**Keywords:** Paracervical block, Diclofenac, Pain relief, Manual vacuum aspiration

## Abstract

**Objective:**

To compare the effectiveness of paracervical block with intramuscular Diclofenac for pain relief during manual vacuum aspiration (MVA) for early pregnancy losses.

**Methodology:**

This was an open label randomized controlled trial. Participants were randomized into two therapeutic groups (A and B) using computer generated numbers. Group A received intramuscular Diclofenac 75 mg. Group B received paracervical block using 1% Lidocaine. Participants were asked to rate their pain level on a continuous 10 cm visual analogue scale (VAS) from 0 (no pain) to 10 (the worst pain ever) within 5 minutes of completing the procedure. Participants' level of satisfaction was assessed within 30 minutes of completing the MVA using Likert scale. Data was analysed using the Statistical Package for Social Sciences (SPSS), Version 20. Test of statistical significance was set at 95% confidence level (P < 0.05). The primary outcome was the level of pain felt by the patient during the procedure (10 cm VAS). Secondary outcomes included patient's satisfaction and adverse events.

**Results:**

There was significant difference in the mean pain level between the intramuscular diclofenac group; 6.5±1.5 (moderate) and those that received paracervical block; 2.3±1.5 (mild), (p-value=0.005). Patients' satisfaction was also better in paracervical block group compared to intramuscular diclofenac group, (p-value=0.005). Both groups were comparable in terms of complications and drug side effects.

**Conclusion:**

Findings from the study suggest that the use of paracervical block compared to intramuscular Diclofenac for pain relief during MVA for incomplete miscarriage significantly reduced pain, improved patients' satisfaction and was comparably safe.

## Introduction

An estimated 15-20% of clinically evident pregnancies and up to 50% of chemically evident pregnancies end in miscarriage.^1^-^5^ About 80-85% of spontaneous miscarriages occur before 12 weeks of gestation.^2^, ^3^ Incomplete miscarriage is a common presentation of early pregnancy losses and a notable cause of maternal morbidity and mortality.^2^, ^6^ ^7^ Manual vacuum aspiration (MVA) remains the preferred method for uterine evacuation following early pregnancy loss due to its safety, effectiveness, low cost and less complication rates.^1^-^4^ It however has a problem of being painful, making the procedure quite distressful to women.^8^-^11^ Studies have shown that up to 97% of women report moderate to severe intensity pain during and immediately following an MVA.^12^

The introduction of MVA has no doubt led to significant improvements in post-abortion care programmes around the world; however, the guidelines for pain control have generally been vague.^13^ Appropriate pain management during and after MVA is one of the most important factors for the success of the procedure and essential to providing woman-centred post-abortion care (PAC).^10^, ^11^ It is also an important determinant of quality of care in PAC services.^10^, ^11^ The goal of pain management is to minimize anxiety and discomfort, and to do so in a way that poses the least possible medication-induced risks and side effects to the woman's health.^11^

Options of pain management during MVA include general anaesthesia, local anaesthesia, narcotic analgesics, non-narcotic analgesics, anxiolytics and verbal psychological support. Available evidence suggests that a combination of non-pharmacological and pharmacological methods may provide sufficient pain control during MVA.^13^ The controversy however lies on the best possible pharmacological method or combination of methods that may offer adequate pain relief with the least possible medication-induced risks and side effects to the woman's health.^11^ The search for an ideal analgesia during MVA, using these methods either alone or in combination has remained a key factor driving various researches on the subject.

The lack of consensus regarding the ideal mode of pain relief during MVA has continued to be a problem in gynaecological practice around the world despite several studies in this regard.^13^ Many gynaecologists use paracervical block (PCB) for uterine interventions; a method that involves injection of local anaesthetic around the cervix to numb nearby nerves, but the effectiveness of this method appears unclear.^13^-^15^ Available evidence seems conflicting on the efficacy of PCB with little or no information about the side effects of the method thus encouraging further research on PCB as a method of pain relief during MVA.^13^

Non-steroidal anti-inflammatory drugs such as diclofenac when administered either orally or parenterally prior to the procedure is also recommended for pain management during MVA as it decreases pain of uterine cramping.^14^ In our centre, women undergoing MVA receive pre-procedure intramuscular Pentazocine 30 mg or Diclofenac 75 mg as single agents, or recently, PCB, with the achievement of varying levels of pain relief and satisfaction during and after the procedure.

The effectiveness of paracervical block and pre-procedure intramuscular Diclofenac has not been fully evaluated in a randomized controlled trial. Therefore, this study set out to compare the effectiveness of pain relief during MVA for early pregnancy losses using paracervical block or intramuscular Diclofenac in an open label randomized controlled trial.

## Methods

The study was an open label randomized controlled trial comparing the effectiveness of pain relief during manual vacuum aspiration for early pregnancy losses using either paracervical block or intramuscular Diclofenac at the University of Abuja Teaching Hospital (UATH), Abuja. The UATH is a 350-bed health facility which provides healthcare services to the inhabitants of Nigeria's Federal capital and its neighbouring states. The study was conducted between 23rd August,2018 and 17th January,2019 Eligible patients for the study were consecutively allocated into one of two therapeutic groups A (intramuscular diclofenac) or B(PCB) from opaque, sealed envelopes labelled 1 to 90 using computer generated random numbers. Each envelope containing a piece of paper designated A-for intramuscular diclofenac group and B-for PCB group was prepared and arranged in ascending order in a box. An envelope was given to each consecutive consenting patient who satisfied the inclusion criteria by the researcher or an assistant. Group A received IM Diclofenac 75 mg, while group B received PCB using 1% Lidocaine. A total of 45 participants were allocated to each group making a total of 90 participants.

All consenting women who presented to the gynaecological emergency with incomplete miscarriage at a gestational age or uterine size not greater than 12 weeks during the study period were consecutively included in the study. The following groups of women were excluded: gestational age or uterine size greater than 12 weeks, clinical signs of uterine sepsis (fever, offensive vaginal discharge or generalised lower abdominal pain), allergy to Diclofenac and/or Lidocaine, haemorrhagic disorder or treatment with anticoagulants and severe illness (sickle cell disease, unconscious patients, significant physical or mental health condition). This information was determined through interview with clients during recruitment as well as by clinical examination and investigations where necessary.

The sample size was determined from the formula below, assuming a difference in mean pain score among groups of 1.5 and using a standard deviation of 2.8^41^


$$\begin{array}{l} \text{n}=\left({\text{Z}}_{\text{α}}+{\text{Z}}_{\text{β}}\right)2\;{\text{S}}^{\text{2}}\\ \;\;\;\;\;{\text{d}}^{\text{2}} \end{array}$$

n = minimum sample size required for each group

Zα - standard normal deviation = 1.96

Zβ - (power of the study to detect significant differences if it exists at 95%) = 1.64

S - standard deviation = 2.8

d - mean difference in pain score = 1.5.



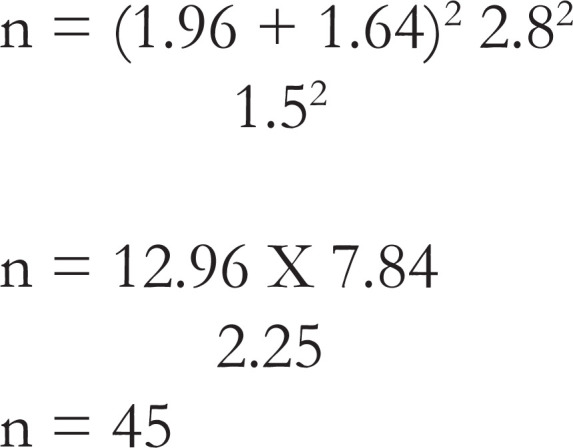



Forty- five women were recruited into each arm of the study to achieve a power of 95% and type 1 error of 0.05 giving a total of 90 women.

### Patient recruitment/ counselling and selection/ measurements

The study was explained to all eligible patients and their written informed consent sought before recruitment. The explanations included a summary of the background information on early pregnancy losses, MVA and its associated complications particularly pain, various modalities of pain management during MVA and their possible advantages and disadvantages and a description of the proposed study intervention. Risks and benefits of participation were discussed. Eligible patients were identified for recruitment. The information sheet was given to patients to read and was read out to those that could not read, following which the researcher or research assistants discussed the study with the patient. The counselling was centred on the need for research in the health sector so as to improve patients' care. Participants' understanding of the explanation about the research was assessed by asking them to reiterate what they understood. Written informed consent was obtained from eligible women and consecutive recruitment of eligible and consenting patients was done.

Structured interviews with the aid of a proforma was conducted at presentation to collect information on socio-demographic characteristics, medical and obstetric history of each participant. Gestational age was determined on best available estimate; either by using the patient's last menstrual period or estimation of gestational age from a first trimester ultrasound scan.

### Intramuscular diclofenac injection

The Diclofenac brand used was Diclofenac sodium 75 mg, marketed with the trade name ‘Olfen’, with the batch number S37348; a product of Oculus pharmacare limited (a Swiss based pharmaceutical company).

Diclofenac was given IM into the upper outer quadrant of the left gluteus muscle, using a 23G hypodermic needle on a 5 ml syringe at a general dose of 75 mg. A latency period of about 10 minutes was allowed before the MVA was commenced to give room for the onset of analgesic action of the drug.

### Paracervical block

PCB was performed with an 18G cannula needle on a 20 ml syringe using a standard dose of 120 mg (12 ml) of 1% Lidocaine per participant. The brand of Lidocaine used was ‘KENLOCAIN’, a product of ANCALIMA Lifesciences Limited, with the batch number 1013001.

To perform the PCB, a sterile Cusco's speculum was introduced into the vagina to expose the cervix. The cervix and vagina were cleansed with antiseptic solution and 2 ml of 1% Lidocaine was injected at 12 0'clock position to a depth of 1.5 - 3 cm. The anterior lip of the cervix was grasped at the injection site with a Vulsellum forceps and a gentle traction applied downwards to stabilize it. 5 ml of 1% Lidocaine was then injected into a depth of 1.5 - 3 cm at 4 and 8 0'clock positions, care being taken to aspirate before injection to avoid intravascular injection. A latency period of about 4 minutes was given before the procedure was commenced to allow for onset of action of the agent.^5^, ^14^

### Manual vacuum aspiration

The manual vacuum aspiration was performed according to standard clinical protocol in the MVA room in the gynaecological ward. The researcher conducted majority of the MVA with the assistance of the research assistants. The pain rating scale, Visual Analogue Scale (VAS) and the Likert scale for level of satisfaction were administered to the participants by the researcher and the research assistants. A first aid box was provided to manage emergency complications.

Each patient was placed in Lithotomy position. Perineum was cleaned with antiseptic solution and draped with sterile towels. The bladder was emptied. A Cusco's speculum was introduced into the vagina and anterior lip of the cervix was held with Vulsellum to straighten the endocervical canal. An appropriate size Karman cannula was inserted into the uterine cavity and connected to the pretested/precharged vacuum syringe and evacuation carried out by rocking and rotating movements of the cannula. The procedure was completed when red foam with gritty sensation were seen and felt respectively and no more tissue entered the cannula. The cannula and Vulsellum were then removed while the speculum were removed after confirming that the bleeding had stopped. The aspirated tissue was examined and sent for histology. The vital signs were monitored quarter hourly until patient was stable for 60 minutes.

The VAS was administered by the researcher or research assistant within 5 minutes of completing the procedure. Participants were asked to rate their pain level felt during the MVA by marking an ‘X’ on the continuous 10 cm VAS line at a point from 0cm (no pain) to 10cm (the worst pain ever), that represented their pain intensity. Using a ruler, the pain score was determined by measuring the distance (in cm) on the 10 cm VAS line between the ‘no pain’ (0 point) and the patient's mark, providing a range of scores from 0-10 cm. Patients' satisfaction was likewise assessed using Likert scale within 30 minutes of completing the procedure; before discharge from the MVA unit by asking the participant to shade the space in front of the options on the scale from very dissatisfied to very satisfied. The completed proforma was analysed by the researcher at the end of the study.

### Data analysis

Data analysis was done using Statistical Package for Social Sciences (SPSS), IBM SPSS statistics for windows, Armonk, NY: IBM Corporation 2011 Version 20. Continuous variables were presented as mean and standard deviation. Categorical i variables were analysed using Chisquare test. Continuous variables were analysed usingg student t-test. Analysis of variance (ANOVA) test was employed where necessary. Statistical significance (P-value) was set at p< 0.05.

## Results

**Figure 1:**
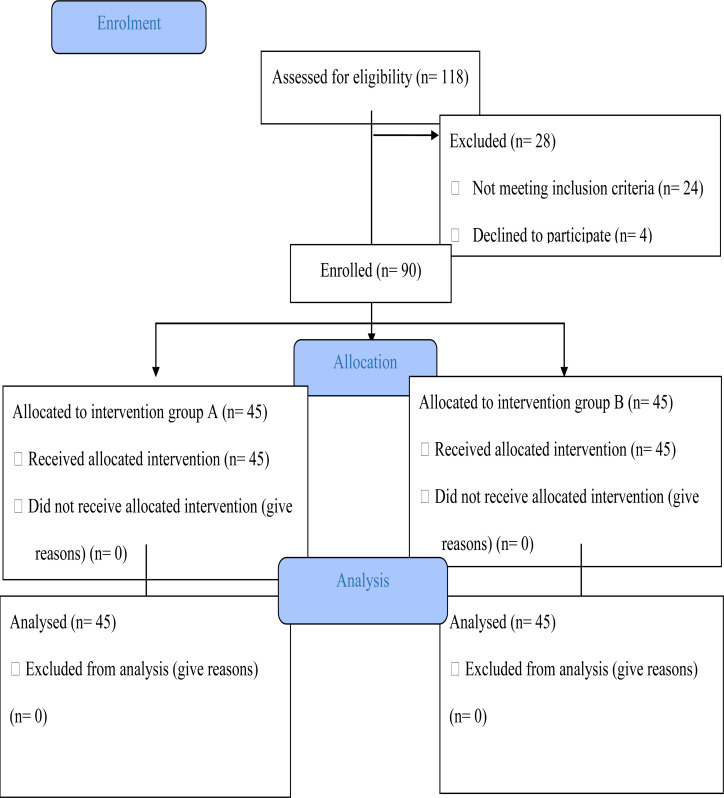
Consort Flow Chart Adapted from CONSORT 2010 flow diagram

A total of 118 participants were assessed for eligibility,24 was excluded for not meeting the inclusion criteria while 4 declined to participate despite meeting inclusion criteria. Therefore, a total of 90 out of 94 women (response rate=95.7%) who met ,the inclusion criteria were randomised and enrolled into the study. Forty-five women were enrolled into the intramuscular Diclofenac group and forty-five into the paracervical block group . All 90 participants completed their visual analogue and Likert's scales and their data were used for the final analysis. (Figure 1)

Both groups were comparable in terms of the mean gestational age, level of menstrual cramps, previous vaginal deliveries and mean body mass index (BMI) of the participants. The mean gestational age for intramuscular Diclofenac group and paracervical block group was 9.0±1.9 versus 9.4±2.0 (p-value =0.099), level of menstrual cramps; 2.8±1.2 versus 2.9±1.3 (p-value =0.824), previous vaginal deliveries; 4.5±2.4 versus 3.4±1.8 (p-value =0.500) and mean BMI; 27.0±3.0 versus 28±4.3 (p-value =0.434). There was significant difference in the means of the maternal age, parity and previous manual vacuum aspiration (MVA), between the two groups. The mean maternal age for intramuscular Diclofenac group and paracervical block group was 33±7.0 versus 30±6.4 (p-value =0.022), parity 3±2.7 versus 2+2.1 (p-value =0.039), and previous MVA 4.2±3.0 versus 2.4±2.0 (p-value =0.033). These are as shown in table 1.

**Table 1:** Socio-demographic and gynaecological characteristics of the participants

Variable	IM Diclofenac	PCB	P value
	n = 45	n = 45	
Mean maternal age (years)	33±7.0	30±6.4	0.022
Mean parity	3±2.7	2±2.1	0.039
Mean gestational age (weeks)	9.0±1.9	9.4±2.0	0.099
**Level of Menstrual cramps**			
Mild	38 (84.4)	36(80.0)	
Moderate	5 (11.1)	7(15.6)	0.824
Severe	2(4.4)	2(4.4)	
**Previous vaginal deliveries**			
No	18(40.0)	19(42.2)	0.500
Yes	27(60.0)	26(57.8)	
**Previous MVA**			
No	27(60.0)	37(82.2)	0.033
Yes	18(40.0)	8(17.8)	
**Mean BMI (kg/m^2^)**	27.0±3.0	28±4.3	0.434

There was significant difference in the mean level of pain perceived by participants in the two groups. The mean level of pain in intramuscular Diclofenac group and paracervical block group were 6.5±1.5 (moderate) versus 2.3±1.5 (mild), (p-value=0.005), respectively. Majority of the participants in paracervical block group ; 41 (91.1%) experienced mild pain compared to only 6 (13.3%) in intramuscular Diclofenac group . Only 4 (8.9%) in paracervical block group experienced moderate pain compared to 31 (68.9%) in intramuscular Diclofenac group. None of the participants in paracervical block group experienced severe pain compared to 8 (17.8%) in intramuscular Diclofenac group . These are as shown in table 2.

**Table 2:** Level of pain by treatment type

Level of pain	IM Diclofenac n = 45 (%)	PCBn = 45 (%)	P value
0 – 4 (mild)	6 (13.3)	41 (91.1)	0.01
5 – 7 (moderate)	31(68.9)	4(8.9)	
8 – 10 (severe)	8(17.8)	0 (0.0)	
Total	45(100.0)	45 (100.0)	
Mean level of pain	6.5± 1.5(Moderate)	2.3±1.5 (Mild) 0.01	

Table 3 shows the level of satisfaction by treatment type. There was a significant difference in the level of satisfaction between the two groups, (p-value=0.005). Paracervical block group had 19(42.2%) being very satisfied, 25(55.6%) were somewhat satisfied, and 1(2.2%) was somewhat dissatisfied. No participant was ‘neither satisfied nor dissatisfied’ and none was very dissatisfied. In intramuscular Diclofenac group , majority of the participants 19(42.2%) were neither satisfied nor dissatisfied, 13(28.9%) were somewhat dissatisfied, 2(4.4%) were very dissatisfied, 10(22.2%) were somewhat satisfied, while only 1(2.2%) was very satisfied.

**Table 3:** Patient's satisfaction by treatment type

Level of satisfaction	IM Diclofenac n = 45 (%)	PCB n = 45 (%)	P value
Very satisfied	1(2.2)	19(42.2)	
Somewhat satisfied	10(22.2)	25(55.6)	
Neither satisfied nor dissatisfied	19(42.2)	0(0.0)	0.01
Somewhat dissatisfied	13(28.9)	1(2.2)	
Very dissatisfied	2(4.4)	0(0.0)	

Table 4 represents the drug side effects in both groups. Majority of the participants in both intramuscular Diclofenac group and paracervical block group ; 44(97.8%) and 43(96.6%), respectively, experienced no drug side effects. Dizziness was observed in only 1(2.2) in both groups. Only 1(2.2%) participant in paracervical block group had nausea. Both groups were comparable in terms of drug side effects p-value=0.603.

**Table 4:** Complications/drug side effects

Side effects	IM Diclofenac n =45(%)	PCB n = 45 (%)	P value
Nausea	0(0.0)	1(2.2)	
Dizziness	1(2.2)	1(2.2)	0.603
None	44(97.8)	43(96.6)	

## Discussion

This study showed a statistically significant difference in mean pain levels between intramuscular Diclofenac group paracervical block group in favour of the paracervical block (PCB) group which means that the use of PCB provided better pain relief during manual vacuum aspiration compared to intramuscular Diclofenac. This finding is comparable to that of Acmaz et al in a study that compared the effect of Paracetamol, Dexketoprofen Trometamol, Lidocaine Spray and Paracervical Block for Pain Relief during Suction Termination of First-Trimester Pregnancy.^16^ In the above study, PCB was found to offer better pain relief compared to Deketoprofen Trometamol; an NSAID just like Diclofenac used in this study. Owolabi et al in a randomized trial of pain relief in termination of pregnancy in South Africa also found that PCB with 1% Lidocaine and intramuscular Diclofenac compared to intramuscular Diclofenac alone, provided better pain relief during and after MVA.^17^ Although the latter study was limited by the fact that two agents were compared to one agent. In a study by Patel et al comparing PCB with general anaesthesia for first trimester cervical dilatation and uterine evacuation, PCB was found to be an effective and safe option for MVA procedure in patients who are at risk of developing general anaesthesia related complications.^18^ Reports from other studies including Cochrane reviews have suggested the efficacy of PCB for pain relief during MVA.^5^, ^13^, ^15^, ^19^, ^20^

This study also found that participants in the paracervical block group were more satisfied with pain relief during MVA than those in the intramuscular diclofenac group . Except for one participant in the paracervical block group who was somewhat dissatisfied, the rest were satisfied with pain relief during the procedure. The reverse was almost the case with the intramuscular diclofenac group where nearly all the participants were dissatisfied with pain relief they got during MVA. This finding is similar to reports from previous studies and may not be unconnected with the mechanisms of action of both methods of pain relief used in this study.^5^, ^13^, ^17^ While paracervical block provides pain relief during MVA by blocking the parasympathetic nerve fibres from S2 - 4 through the Frankenhauser's plexus which innervates the cervix and lower uterine body , intramuscular diclofenac blocks the sympathetic fibres T10 - L1 via the inferior hypogastric nerve which enter the uterus at the uterosacral and utero-ovarian ligaments, and ovarian plexuses which enter at the cornua. The latter acts by reducing the formation of prostaglandins, with their effects extending to the cervix.^11^,^13^ From the foregoing paracervical block may therefore tend be more effective in controlling pain from both uterine cramping and cervical dilatation while intramuscular diclofenac pain control effects would be much more for uterine cramping and lesser for cervical dilatation.

There were no notable complications among the participants in either of the groups in this study. Both groups were comparable in terms of drug side effects. Only one participant in group A complained of nausea following intramuscular Diclofenac injection, while two participants in group B complained of nausea and dizziness, respectively. This is similar to the findings of Natalia et al in “Comparison of Effectiveness of Pain Management during Manual Vacuum Aspiration Using Single-Agent Analgesic and Combination: A Randomized Double-Blind Controlled Trial”, and that of Owolabi et al in a randomized trial of pain relief in termination of pregnancy in South Africa.^16^, ^17^

There was no statistically significant difference between the two groups in terms of the mean gestational age, level of menstrual cramps, previous vaginal deliveries and mean body mass index (BMI) of the participants. Both groups differed significantly in terms of previous manual vacuum aspiration, mean maternal age and mean parity of the participants. The diclofenac group had higher mean parity and a higher prevalence of prior MVA than those in the paracervical block group. The implication is that these results may be a conservative estimate of the pain level in the diclofenac group given that they may be expected to have a higher pain tolerance due to their prior experiences. This further supports the current findings that paracervical block provided better pain control and satisfaction when compared to intramuscular diclofenac.

## Conclusion

Findings from the study suggest that the use of paracervical block compared to intramuscular Diclofenac for pain relief during manual vacuum aspiration for incomplete miscarriage significantly reduced pain, improved patients' satisfaction and was comparably safe.

The adoption of paracervical block as a preferred method of analgesia may offer an opportunity for effective pain management and improved patient satisfaction for women undergoing MVA for early pregnancy losses in our Gynaecological Emergency units.

## Data Availability

The data sets generated during and/or analysed during the current study are available from the corresponding author on reasonable request.
